# Respiratory mechanics measured by forced oscillation technique in rheumatoid arthritis-related pulmonary abnormalities: frequency-dependence, heterogeneity and effects of smoking

**DOI:** 10.1186/s40064-016-1952-8

**Published:** 2016-03-15

**Authors:** Risa Sokai, Satoru Ito, Shingo Iwano, Akemi Uchida, Hiromichi Aso, Masashi Kondo, Naoki Ishiguro, Toshihisa Kojima, Yoshinori Hasegawa

**Affiliations:** Department of Respiratory Medicine, Nagoya University School of Medicine, Nagoya, 466-8550 Japan; Department of Radiology, Nagoya University School of Medicine, Nagoya, 466-8550 Japan; Department of Clinical Laboratory, Nagoya University School of Medicine, Nagoya, 466-8550 Japan; Department of Orthopedic Surgery and Rheumatology, Nagoya University School of Medicine, Nagoya, 466-8550 Japan

**Keywords:** Airway, Forced oscillation technique, Impedance, Interstitial lung disease, MostGraph, Rheumatoid arthritis

## Abstract

Rheumatoid arthritis (RA)-related pulmonary disorders specifically airway abnormalities and interstitial pneumonia (IP) are important extra-articular manifestations. The forced oscillation technique (FOT) is a useful method to assess respiratory impedance, respiratory resistance (Rrs) and reactance (Xrs), at different oscillatory frequencies during tidal breathing. The aim of this study was to characterize the respiratory mechanics of patients with RA and to relate them to parameters of the pulmonary function test and findings of chest CT images. Respiratory impedance of RA patients (n = 69) was measured as a function of frequency from 4 to 36 Hz using the FOT device and compared with that of healthy subjects (n = 10). Data were retrospectively reviewed. Patients were female-dominant (60.9 %) and 95.7 % had abnormal CT findings including airway and parenchymal abnormalities. Thirty-seven of 69 patients (53.6 %) were smokers. Rrs was significantly frequency-dependent in RA patients but not in the healthy subjects. Xrs were significantly frequency-dependent in both RA and healthy groups. Rrs was significantly higher during an expiratory phase in both RA and healthy groups. Xrs was significantly lower (more negative) during an expiratory phase than that during an inspiratory phase in RA patients but not in healthy subjects. Xrs of the RA group was significantly more negative than that of the normal control. There was no difference in impedance parameters between the airway lesion dominant (n = 27) and IP dominant groups (n = 23) in the RA group. The impedance parameters of the RA group significantly correlated with most parameters of the pulmonary function test. In pulmonary function test results, % of the predicted value for forced expiratory flow from 25 to 75 % of forced vital capacity was significantly lower and % of the predicted value for diffusing capacity of the lung for carbon monoxide was higher in the airway lesion dominant group than those in the IP dominant group. Krebs von den Lungen-6, a serum indicator of IP, was significantly higher in the IP group than that in the airway lesion dominant group. Taken together, the impedance results reflect abnormalities in pulmonary functions and structures in patients with RA.

**Electronic supplementary material:**

The online version of this article (doi:10.1186/s40064-016-1952-8) contains supplementary material, which is available to authorized users.

## Background

Rheumatoid arthritis (RA) is a systemic inflammatory disease associated with extra-articular diseases including pulmonary diseases (Perez et al. 1998; Turesson et al. 2003; Brown 2007). RA-related pulmonary disorders, specifically interstitial pneumonia (IP) and airway abnormalities, are recognized as an important extra-articular manifestation because they are responsible for a significant portion of the mortality (Brown 2007; Olson et al. 2011; Tsuchiya et al. 2011). Computed tomography (CT) of the lung and pulmonary function tests have been used widely to manage the pulmonary abnormalities of patients with RA (Cortet et al. 1997; Fuld et al. 2003; Biederer et al. 2004; Tanaka et al. 2004; Mori et al. 2008, 2011).

The forced oscillation technique (FOT) is an accurate method to assess respiratory mechanics from input impedance measurements (Dubois et al. 1956; Grimby et al. 1968; Michaelson et al. 1975). This method is less dependent on patient effort than spirometry. Another benefit of FOT is that it enables measurement of both inspiratory and expiratory parameters during tidal breathing (Cauberghs and Van de Woestijne 1992; Peslin et al. 1992; Dellaca et al. 2004; Kanda et al. 2010; Paredi et al. 2010; Fujii et al. 2015). Measurement of respiratory system impedance (Zrs), respiratory resistance (Rrs) and reactance (Xrs), has been used successfully to assess respiratory functions of normal subjects and patients with respiratory diseases such as asthma, chronic obstructive pulmonary disease (COPD), and interstitial lung disease (van Noord et al. 1989; Dellaca et al. 2004; Kanda et al. 2010; Paredi et al. 2010; Ohishi et al. 2011; Ito et al. 2012; Miranda et al. 2013; Shirai et al. 2013; Sugiyama et al. 2013; Fujii et al. 2015; Hasegawa et al. 2015). Rrs reflects the extent of airflow obstruction (Di Mango et al. 2006; Hasegawa et al. 2015). Xrs is determined by the elastic properties of the respiratory system at the lowest frequency and the inertive properties at higher frequencies (Oostveen et al. 2003). It is expected that FOT will be able to identify respiratory abnormalities in patients with RA that are not detectable by spirometric examinations (Faria et al. 2012). However, the Zrs assessed by this technique has not been fully evaluated in patients with RA-related pulmonary diseases yet.

The purpose of the present study was to characterize the respiratory mechanics measured by FOT in patients with RA and to relate them to parameters of pulmonary function tests and findings of chest CT images. In addition, frequency-dependent and within-breath behavior of the respiratory mechanics were also evaluated.

## Results

### Clinical characteristics and pulmonary function test results

The characteristics and laboratory and pulmonary function test results of the 69 patients with RA shown in Table 1. 88.2 % were positive for anti-cyclic citrullinated peptide (anti-CCP) antibody (≥4.5 U/ml), and 86.3 % were positive for rheumatoid factor (RF) (≥20.0 U/ml). Next, the characteristics and pulmonary function test results of the airway lesion predominant and IP predominant groups were compared. The predominant CT patterns were classified as: airway lesion predominant (n = 27, 39.1 %), IP predominant (n = 23, 33.3 %), mixed pattern of airway abnormalities and IP (n = 5, 7.2 %), other patterns (n = 11, 15.9 %), or no abnormal findings (n = 3, 4.3 %). Krebs von den Lungen-6 (KL-6), a serum indicator of IP, was significantly higher in the IP group than those in the airway lesion dominant group (Table 1). Pulmonary function test results show that % of the predicted value for diffusing capacity of the lung for carbon monoxide (%DL_CO_) and residual volume (RV)/total lung capacity (TLC) ratio were significantly higher and % of the predicted value for forced expiratory flow from 25 to 75 % of forced vital capacity (FVC) (%FEF_25–75_), which reflects small airway diseases in RA (Mori et al. 2008; Mori et al. 2011), was lower in the airway lesion dominant group than that in the IP group (Table 1). Thirty-seven of 69 patients (53.6 %) were smokers, but there were no significant difference in smoking history between the airway lesion dominant and IP groups (Table 1).

**Table 1:** Clinical characteristics and pulmonary function test results of investigated subjects

Subjects	Total, n = 69	Airway, n = 27	IP, n = 23	*P* value
Age, years (range)	65.5 ± 10.1 (39–86)	65.6 ± 9.4 (39–80)	62.5 ± 9.6 (39–78)	0.253
Sex, male/female	27/42	8/19	14/9	0.053
Height, cm	157.2 ± 10.4	155.2 ± 8.5	162.4 ± 12.1	0.016*
Weight, kg	57.9 ± 13.5	55.2 ± 12.9	62.3 ± 14.3	0.071
BMI	23.3 ± 4.6	22.9 ± 5.3	23.4 ± 3.4	0.729
Current/ex/never smokers	9/28/32	2/10/15	4/12/7	0.177
Pack-years (range)	45.8 ± 34.8 (0.8-147.0)	41.0 ± 34.2 (0.8–105.0)	47.3 ± 34.7 (1.2–147.0)	0.639
Duration of RA, years	12.5 ± 10.1	14.0 ± 9.2	8.5 ± 6.3	0.019*
Anti-CCP Ab, U/ml	187.3 ± 274.2 (n = 51)	221.6 ± 422.6 (n = 17)	222.8 ± 243.6 (n = 19)	0.936
RF, IU/ml	254.3 ± 514.7 (n = 51)	218.8 ± 445.3 (n = 19)	208.3 ± 249.6 (n = 16)	0.892
KL-6, U/ml	492.7 ± 336.2 (n = 58)	394.3 ± 270.9 (n = 22)	646.0 ± 388.7 (n = 21)	0.018*
SP-D, ng/ml	68.6 ± 31.0 (n = 19)	47.2 ± 22.3 (n = 5)	75.8 ± 29.5 (n = 10)	0.081
LDH, IU/ml	214.5 ± 44.7	218.1 ± 46.0	213.7 ± 52.1	0.750
%VC	97.6 ± 15.4	95.6 ± 16.6	95.0 ± 13.9	0.938
%FVC	99.9 ± 17.7	97.2 ± 19.6	97.9 ± 16.2	0.884
%FEV_1_	87.8 ± 19.1	81.8 ± 20.0	89.3 ± 19.3	0.182
FEV_1_/FVC, %	71.1 ± 10.3	67.9 ± 11.4	74.5 ± 7.8	0.025*
FEF_25–75_/FVC, %	51.9 ± 25.8	44.0 ± 25.1	62.1 ± 25.2	0.016*
%FEF_25–75_	52.6 ± 25.7	42.3 ± 23.1	63.1 ± 24.8	0.004*
%TLC	102.2 ± 15.3	102.9 ± 14.4	97.7 ± 16.2	0.233
%RV	100.1 ± 22.1	104.7 ± 22.0	92.5 ± 21.3	0.054
RV/TLC %	37.6 ± 7.2	39.6 ± 7.9	34.1 ± 5.0	0.006*
%DL_CO_	97.0 ± 22.0	104.9 ± 15.9	80.1 ± 20.0	<0.001*
%DL_CO_/V_A_	99.7 ± 26.3	107.3 ± 18.2	84.1 ± 25.8	<0.001*

Values are mean ± SD. Values were compared using t-test, Chi square test, or Fisher’s exact test

*Anti-CCP Ab* anti-cyclic citrullinated peptide antibody, *RF* rheumatoid factor, *KL-6* Krebs von den Lungen, a serum indicator of interstitial pneumonia, *SP-D* surfactant protein-D, *FEV*_*1*_ forced expiratory volume in 1 s, *FVC* forced vital capacity, *VC* vital capacity, *FEF*_*25–75*_ forced expiratory flow from 25 to 75 % of FVC, *TLC* total lung capacity, *RV* residual volume, *DL*_*CO*_ diffusing capacity of the lung for carbon monoxide, *V*_*A*_ alveolar volume

* Significantly different (*P* < 0.05) between the airway lesion dominant and IP dominant groups

### CT findings

Representative CT images of airway lesion dominant and IP dominant patterns are shown in Additional file 1: Figure S1. The characteristics, frequency, and grades of CT findings in all 69 cases, airway lesion predominant group (n = 27), and IP predominant group (n = 23) are shown in Table 2. Grades and frequency of CT findings of the airway lesion dominant and IP dominant groups were compared (Table 2). Characteristic CT findings of IP such as ground grass opacity, reticulation, honey combing, and traction bronchiectasis were more frequent in the IP dominant group. On the other hand, findings of bronchiectasis/bronchiolectasis and bronchiolar abnormality were more frequent in the airway lesion dominant group.

**Table 2:** CT findings

Findings, n (%)	Total, n = 69	Airway, n = 27	IP, n = 23	*P* value
Airspace consolidation	25 (36.2 %)	15 (55.6 %)	5 (21.7 %)	
Grade, median (range)	0 (0–2)	1 (0–1)	0 (0–1)	0.034*
Ground grass opacity	31 (44.9 %)	5 (18.5 %)	18 (78.3 %)	
Grade, median (range)	0 (0–4)	0 (0–2)	1 (0–4)	<0.001*
Reticulation	41 (59.4 %)	11 (40.7 %)	20 (87.0 %)	
Grade, median (range)	1 (0–3)	0 (0–1)	1 (0–3)	<0.001*
Bronchovascular bundle thickening	1 (1.4 %)	1 (3.7 %)	0	
Grade, median (range)	0 (0–1)	0 (0–1)	0 (0–0)	0.377
Honeycombing	10 (14.5 %)	0	9 (39.1 %)	
Grade, median (range)	0 (0–3)	0 (0–0)	0 (0–3)	<0.001*
Nodules	49 (71.4 %)	26 (96.3 %)	10 (43.5 %)	
Grade, median (range)	1 (0–4)	1 (0–4)	0 (0–1)	<0.001*
Emphysema	20 (29.0 %)	2 (7.4 %)	13 (56.5 %)	
Grade, median (range)	0 (0–3)	0 (0–2)	1 (0–3)	<0.001*
Bullae	24 (34.8 %)	4 (14.8 %)	13 (56.5 %)	
Grade, median (range)	0 (0–1)	0 (0–1)	1 (0–1)	<0.001*
Bronchiectasis or bronchiolectasis	23 (33.3 %)	19 (70.4 %)	0	
Grade, median (range)	0 (0–2)	1 (0–2)	0 (0–0)	<0.001*
Traction bronchiectasis	20 (29.0 %)	3 (11.1 %)	17 (73.9 %)	<0.001*
Crazy-paving appearance	2 (2.9 %)	0	2 (8.7 %)	0.207
Tree-in-bud sign	6 (8.7 %)	5 (18.5 %)	0	0.054
Architectural distortion	14 (20.3 %)	4 (14.8 %)	8 (34.8 %)	0.183
Pulmonary artery enlargement	2 (2.9 %)	0	1 (4.3 %)	0.460
Esophageal dilatation	15 (21.7 %)	4 (14.8 %)	8 (34.8 %)	0.183
Lymph node enlargement	16 (23.2 %)	6 (22.2 %)	5 (21.7 %)	1
Pleural or pericardial effusion or thickening	16 (23.2 %)	6 (22.2 %)	5 (21.7 %)	1
Bronchiolar abnormality	33 (47.8 %)	25 (92.6 %)	3 (13.0 %)	<0.001*

*Airway* airway lesion dominant pattern, *IP* interstitial pneumonia dominant pattern

* Significantly different (*P* < 0.05) between airway lesion and IP dominant groups (Mann–Whitney test or Fisher’s exact test)

### Respiratory impedance of RA

**Fig. 1:**
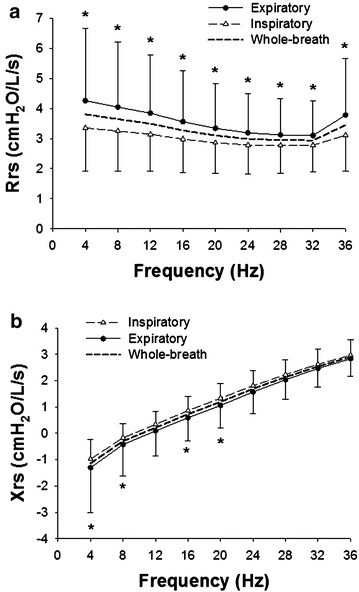
Frequency dependences of respiratory impedance, respiratory resistance (Rrs) and reactance (Xrs) at 4–36 Hz, during a whole breath, inspiratory phase and expiratory phase, were examined. The Rrs (**a**) and Xrs (**b**) of all rheumatoid arthritis (RA) cases (n = 69) are shown. Values during inspiratory and expiratory phases are mean ± SD (cmH_2_O/L/s). Averages of Rrs and Xrs during a whole breath are also shown (*dashed lines*). *Significant difference (*P* < 0.05) between inspiratory and expiratory phases by two-way repeated measure ANOVA, followed by Bonferroni test for post hoc analysis

Rrs and Xrs results at a given frequency of all 69 patients are shown in Fig. 1. The Rrs values during a whole breath, inspiratory phase, and expiratory phase were significantly frequency-dependent (*P* < 0.001) and gradually decreased as a function of frequency from 4 to 32 Hz but increased at 36 Hz (Fig. 1a). Rrs values were significantly higher during the expiratory phase than during the inspiratory phase (*P* < 0.001) at all frequencies (Fig. 1a). Xrs values during a whole breath, inspiration, and expiration were also significantly increased as a function of frequency (Fig. 1b). Expiratory Xrs values were significantly lower than inspiratory Xrs (*P* = 0.004) specifically at lower frequencies (4–20 Hz) (Fig. 1b).

High prevalence (53.6 %) of smoking history in our RA cohort (Table 1) is an important cofounding factor. Thus, we compared the Zrs results of smokers (n = 37) with those of never smokers (n = 32). However, there was no difference in Rrs or Xrs during a whole breath between ever and never smokers (Additional file 2: Figure S2A and S2B). Moreover, differences between inspiratory and expiratory phases (Δ) in Xrs (ΔXrs) calculated as mean inspiratory values minus mean expiratory values were not statistically significantly different between the groups (Additional file 2: Figure S2C).

### Respiratory impedance of healthy control subjects

**Fig. 2:**
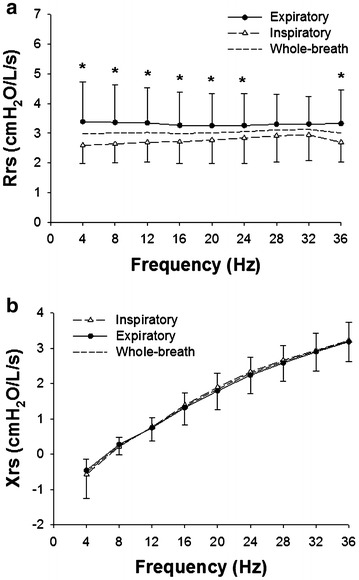
Frequency dependences of the Rrs (**a**) and Xrs (**b**) of the healthy subjects (n = 10) are shown. Values during inspiratory and expiratory phases are mean ± SD (cmH_2_O/L/s). Averages of Rrs and Xrs during a whole breath are also shown (*dashed lines*). *Significant difference (*P* < 0.05) between inspiratory and expiratory phases by two-way repeated measure ANOVA, followed by Bonferroni test for post hoc analysis

Next, we re-analyzed the Zrs results at a given frequency of the healthy control group of our previous study (age: 24–59 years, n = 10) (Uchida et al. 2013). The biometric and spirometric characteristics of the healthy control group are shown in Additional file 3: Table S1. Rrs was not significantly frequency-dependent during a whole breath, inspiratory phase, or expiratory phase (Fig. 2a) different from that in the RA group (Fig. 1a). Rrs values were significantly higher during the expiratory phase than during the inspiratory phase (*P* < 0.001) at most frequencies (Fig. 2a). Xrs values during a whole breath, inspiration, and expiration were also significantly increased as a function of frequency (Fig. 2b). There was no difference in Xrs between inspiratory and expiratory phases (Fig. 2b).

### Comparison of impedance between RA and control groups

**Fig. 3:**
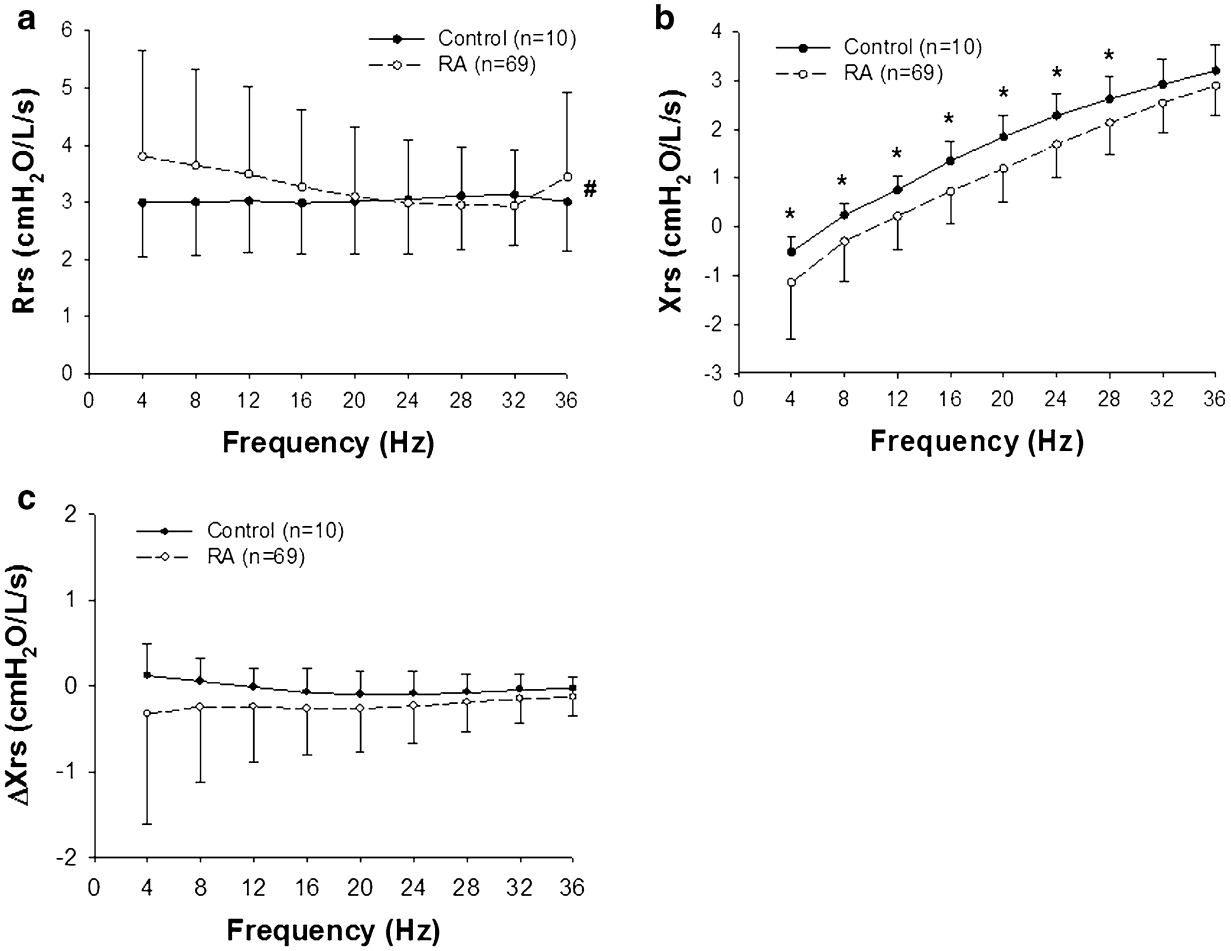
Rrs (**a**) and Xrs (**b**) during a whole breath in the RA (n = 69) and healthy control groups (n = 10) are compared. **c** Differences between inspiratory and expiratory phases (Δ) in Xrs (ΔXrs) calculated as mean inspiratory values minus mean expiratory values are also compared. Values are mean ± SD (cmH_2_O/L/s). *Significant difference (*P* < 0.05) between inspiratory and expiratory phases by two-way repeated measure ANOVA, followed by Bonferroni test for post hoc analysis. ^#^There was a significant interaction in the Rrs curves between the group (either the RA or healthy control) and frequency (*P* < 0.001)

Next, we compared the Zrs results of the RA and healthy control groups. Rrs was significantly frequency-dependent in the RA group (Fig. 1a) but not in the healthy control group (Fig. 2a). As a results, there was a significant interaction in the Rrs curves (*P* < 0.001) between group (either the RA or healthy control) and frequency by two-way repeated-measure analysis of variance (ANOVA) during a whole breath (Fig. 3a), inspiration, and expiration. There was no significant difference between the groups in the mean values of Rrs during a whole breath (Fig. 3a), inspiration, and expiration. Xrs during a whole breath of the RA group was significantly lower (more negative) (*P* = 0.018) than that of the control group (Fig. 3b). Similarly, Xrs during inspiratory and expiratory phases was significantly more negative than that of the control group. ΔXrs was not statistically significantly different between the groups (Fig. 3c).

### Comparison of impedance between airway lesion and IP dominant groups in RA

**Fig. 4:**
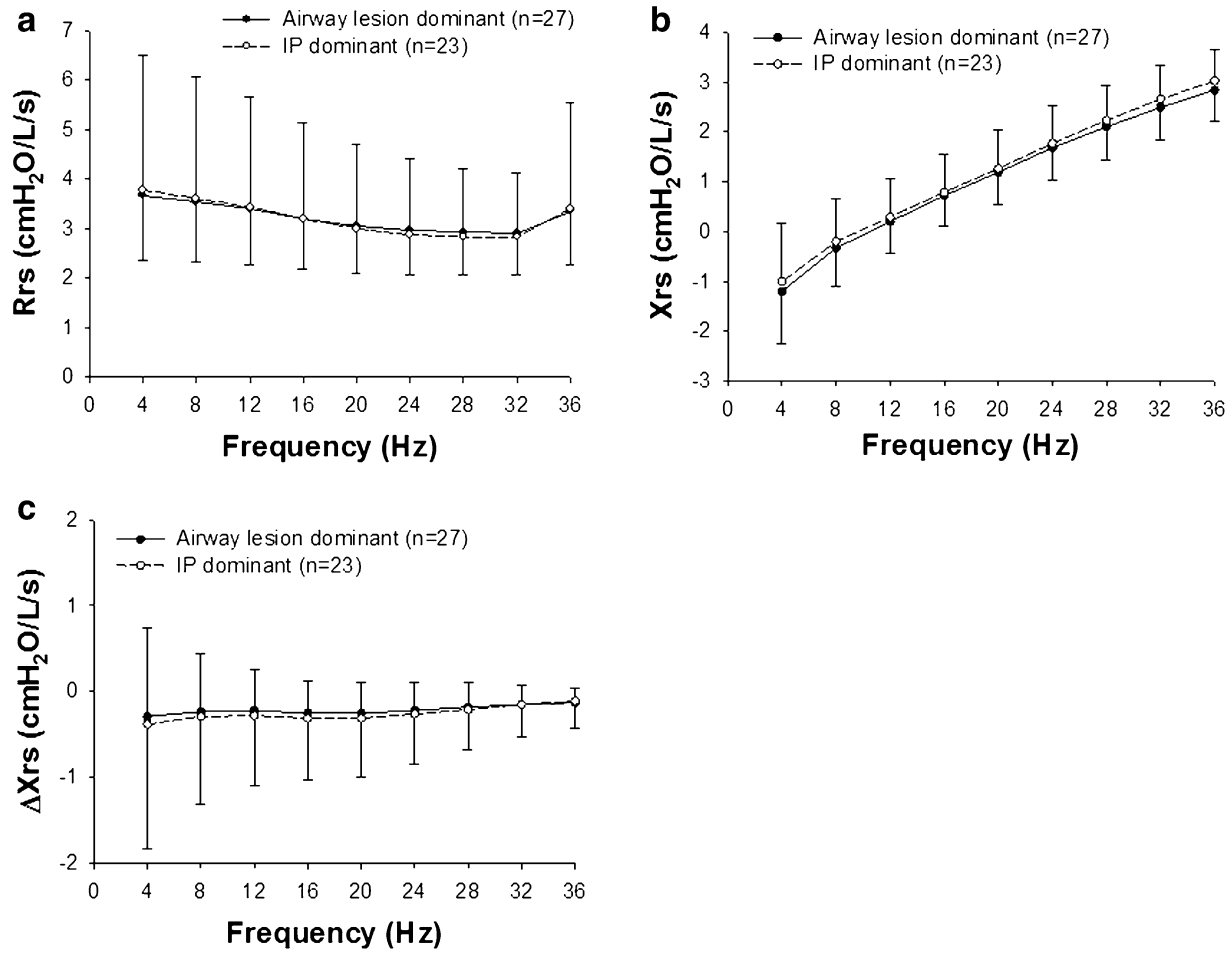
Rrs (**a**) and Xrs (**b**) during a whole breath in the airway lesion dominant (n = 27) and interstitial pneumonia (IP) dominant groups (n = 23) are shown. **c** ΔXrs values are also compared in the airway lesion dominant (n = 27) and IP dominant groups. Values are mean ± SD (cmH_2_O/L/s) and compared by two-way repeated measure ANOVA, followed by Bonferroni test for post hoc analysis

Next, we compared the Zrs results of the airway lesion dominant and IP dominant groups in RA patients. During a whole breath, inspiratory phase, and expiratory phase, Rrs and Xrs were significantly frequency-dependent (*P* < 0.001) in both groups. However, there was no significant difference between the groups in values of Rrs or Xrs (Fig. 4a, b). ΔXrs was not statistically significantly different either (Fig. 4c).

### Correlations between impedance and pulmonary function test results

**Fig. 5:**
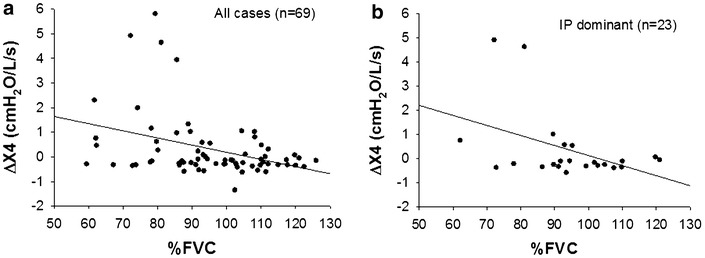
Correlations between % of the predicted value for forced vital capacity (%FVC) and ΔXrs at 4 Hz (ΔX4) in all cases (n = 69, **a**) and IP dominant group (n = 23, **b**)

Correlations between parameters of the Zrs and pulmonary function test are shown in Table 3. Rrs values at the lowest (4 Hz, R4), middle (20 Hz, R20), and highest (36 Hz, R36) frequencies during a whole breath were selected for analysis. ΔXrs at low frequencies is increased by expiratory flow limitation in patients with COPD (Dellaca et al. 2004) and becomes negative in restrictive disorder in patients with interstitial lung diseases (Sugiyama et al. 2013; Fujii et al. 2015). Therefore, Xrs and ΔXrs at the lowest frequency (4 Hz, X4 and ΔX4, respectively) were selected for analysis. ΔX4 significantly negatively correlated with %FVC, % of the predicted value for forced expiratory volume in 1 s (%FEV_1_), and of the predicted value for TLC (%TLC). Relationships between %FVC and ΔX4 for all cases and the IP dominant group are shown (Fig. 5a, b). ΔX4 also inversely correlated with  %FVC in the IP group alone (r = −0.426, *P* < 0.05, Fig. 5b).

**Table 3:** Correlation between parameters of impedance and pulmonary function tests

	R4	R20	R36	X4	ΔX4
Height					
R	−0.323	−0.454	−0.404	0.220	0.127
*P*	0.007*	<0.001*	<0.001*	0.070	0.300
VC					
R	−0.380	−0.429	−0.412	0.480	0.017
*P*	0.001*	<0.001*	<0.001*	<0.001*	0.892
%VC					
R	−0.140	0.022	−0.044	0.497	−0.229
*P*	0.251	0.859	0.717	<0.001*	0.059
FVC					
R	−0.424	−0.456	−0.445	0.519	−0.032
*P*	<0.001*	<0.001*	<0.001*	<0.0001*	0.7947
%FVC					
R	−0.201	−0.018	−0.091	0.517	−0.312
*P*	0.098	0.884	0.457	<0.001*	0.009*
FEV_1_					
R	−0.428	−0.395	−0.418	0.506	−0.024
*P*	<0.001*	<0.001*	<0.001*	<0.001*	0.847
%FEV_1_					
R	−0.220	−0.018	0.109	0.485	−0.240
*P*	0.069	0.883	0.375	<0.001*	0.047*
FEV_1_/FVC					
R	−0.136	−0.051	−0.103	0.080	0.038
*P*	0.264	0.681	0.402	0.515	0.758
FEF_25–75_					
R	−0.299	−0.235	−0.275	0.314	−0.002
*P*	0.013*	0.052	0.022*	0.009*	0.985
%FEF_25–75_					
R	−0.176	−0.069	−0.124	0.240	−0.006
*P*	0.147	0.573	0.310	0.047*	0.964
FEF_25–75_/FVC					
R	−0.127	−0.042	−0.092	0.077	0.039
*P*	0.298	0.730	0.454	0.528	0.752
TLC					
R	−0.380	−0.442	−0.421	0.454	0.028
*P*	0.0013*	0.0001*	0.0003*	<0.001*	0.817
%TLC					
R	−0.148	0.051	−0.033	0.501	−0.301
*P*	0.225	0.675	0.787	<0.001*	0.012*
RV					
R	−0.162	−0.227	−0.205	0.099	0.086
*P*	0.183	0.061	0.091	0.416	0.481
%RV					
R	−0.108	−0.012	−0.057	0.170	−0.159
*P*	0.379	0.920	0.640	0.162	0.192
RV/TLC					
R	0.196	0.195	0.194	−0.370	0.079
*P*	0.107	0.109	0.111	0.002*	0.518
%DL_CO_					
R	0.064	0.088	0.077	−0.002	0.011
*P*	0.605	0.471	0.532	0.986	0.929

Correlation coefficient (R) and significance (*P*) between parameters of impedance and height or pulmonary function test results. R4, R20, and R36, respiratory resistance (Rrs) at 4, 20, and 36 Hz during a whole breath, respectively; X4, respiratory reactance (Xrs) at 4 Hz during a whole breath; ΔX4, difference between mean inspiratory and mean expiratory phases (Δ) in Xrs (ΔXrs) at 4 Hz

* Statistically significant relationship (*P* < 0.05)

### Association between CT findings and impedance results

Next, we examined whether values of the Zrs parameters were affected by the existence of RA-related pulmonary abnormalities based on CT findings. Due to rare prevalence (prevalence ≤ 2 in Table 2), bronchovascular bundle thickening (n = 1), crazy-paving appearance (n = 2), and pulmonary artery enlargement (n = 2) in CT findings were excluded for the analysis. X4 during a whole breath was significantly lower (more negative) in patients with esophageal dilatation than those without (−1.66 ± 1.11 cmH_2_O/L/s vs. −1.00 ± 1.13 cmH_2_O/L/s, *P* < 0.05). ΔX4 was significantly higher in patients with architectural distortion than those without (1.05 ± 2.27 cmH_2_O/L/s vs. 0.14 ± 0.83 cmH_2_O/L/s, *P* < 0.05). Values of the Rrs parameters were not significantly affected by the existence of either CT finding.

## Discussion

The main findings of the present study were that in patients with RA: (1) Rrs and Xrs values were significantly dependent on frequency and differed between expiratory and inspiratory phases, (2) Xrs values and frequency-dependent behavior in Rrs were significantly different from those of the healthy subjects, (3) impedance parameters significantly correlated with most parameters of the pulmonary function test, (4) impedance results were not significantly different between the airway lesion dominant and IP dominant groups, and (5) KL-6, %DL_CO_, RV/TLC, %FEF_25–75_, and FEV_1_/FVC were significantly different between the airway lesion dominant and IP dominant groups. It has been reported that KL-6 is a useful serum marker reflecting IP specifically pulmonary fibrosis including in RA-related IP (Kinoshita et al. 2004). Parameters of the pulmonary function test such as %DL_CO_, FEV_1_/FVC, and %FEF_25–75_ have been used to evaluate functional impairment in pulmonary manifestations associated with RA (Fuld et al. 2003; Mori et al. 2008, 2011). Our findings are consistent with results of those previous reports. In contrast, Rrs, Xrs, or ΔXrs was not significantly different between the airway lesion dominant and IP dominant groups unexpectedly probably due to heterogeneity and large variability as one of characteristics of RA-related pulmonary abnormalities. To our knowledge, however, this is the first study to characterize the respiratory impedance measured by FOT and relate it to pulmonary functions and CT findings in patients with RA-related pulmonary diseases.

One of the advantages of FOT is the ability to evaluate Zrs over a range of frequencies (Dubois et al. 1956; Grimby et al. 1968; Michaelson et al. 1975; Hantos et al. 1992; Ito et al. 2007; Bates et al. 2011; Tanimura et al. 2014). We examined the Rrs and Xrs data at a given frequency between 4 and 36 Hz and found that the Rrs and Xrs of the RA group were significantly frequency-dependent in all cases (Fig. 1) and airway lesion dominant and IP dominant groups (Fig. 4). It is generally known that Xrs increases from negative values to positive values in a frequency-dependent manner both in healthy subjects and patients with respiratory diseases (Oostveen et al. 2003; Bates et al. 2011) as found in the present study. In contrast, Rrs in healthy subjects was not significantly frequency dependent (Fig. 2). As a result, there was a significant interaction between the frequency and group when the Rrs values of RA and normal subjects were compared (Fig. 3a). The frequency-dependence of Rrs reflects the inhomogeneity in gas flow in the respiratory system specifically during bronchoconstriction as well as in patients with COPD and restrictive abnormality due to interstitial lung disease (van Noord et al. 1989; Pride 1992; Lutchen et al. 1996). Taken together, the results suggest that the frequency-dependence in Rrs in medium frequency range derives from both airway and parenchymal abnormalities in patients with RA.

The within-breath behavior of the Zrs results showed that Rrs was significantly higher during the expiratory phase than that during the inspiratory phase in RA (Fig. 1a). Expiratory Xrs was slightly but significantly lower than inspiratory Xrs at lower frequencies in the RA group (Fig. 1b). However, there was no difference in ΔXrs between the airway lesion dominant and IP dominant groups (Fig. 4c). Dellaca et al. reported that ΔXrs at low frequencies is beneficial for detecting expiratory flow limitation in patients with COPD (Dellaca et al. 2004). They analyzed individual respiratory cycles and proposed 2.8 cmH_2_O/L/s of ΔX5 as an optimal threshold value of for expiratory flow limitation (Dellaca et al. 2004). In contrast to their analysis, we calculated the average of respiratory cycles during the Zrs measurements for Δ Xrs analysis as reported by other groups (Kanda et al. 2010; Paredi et al. 2010; Mori et al. 2013; Sugiyama et al. 2013). In the present study, ΔX4 values were above 2.8 cmH_2_O/L/s in four RA cases including two cases in the IP dominant group (Fig. 5) but not in the healthy subjects. Kanda et al. demonstrated that expiratory R5 was significantly higher than inspiratory R5, but there was no significant difference in ΔXrs at 5 Hz (ΔX5) between the expiratory and inspiratory phases in the healthy subjects (Kanda et al. 2010). Similarly, we also found that Rrs was higher during the expiratory phase, but there was no significant difference in Xrs between the expiratory and inspiratory phases in the healthy subjects (Fig. 2). Interestingly, previous studies have demonstrated that inspiratory X5 of IP was significantly lower than expiratory X5 different from the findings in COPD (Mori et al. 2013; Sugiyama et al. 2013). In the present study, ΔX4 values of the airway lesion dominant and IP dominant groups were not different (Fig. 4c), inconsistent with findings in those previous reports (Mori et al. 2013; Sugiyama et al. 2013). There was a weak but significant negative correlation between ΔX4 and %FVC or %TLC, an indicator of IP severity (Table 3; Fig. 5). Moreover, ΔX4 with architectural distortion in the CT findings was significantly higher than that without. Although the origin of ΔXrs is still uncertain, high ΔXrs values at low frequencies may derive from the presence of airway abnormalities, including expiratory flow limitations and decreases in lung volume in our cohort. It has been reported that small airway abnormalities are involved in RA patients even when the CT findings show IP dominant patterns or normal shadow (Mori et al. 2011; Faria et al. 2012) different from idiopathic IP and other collagen vascular diseases-related IP.

Impedance parameters, R4, R20, R36, and X4, significantly correlated with most parameters of the pulmonary function test (Table 3). X4 significantly correlated with parameters for restrictive abnormalities (VC, %VC, FVC, %FVC, TLC, and %TLC), consistent with previous findings in IP (Fujii et al. 2015). These findings indicate that X4 values may be useful to detect lung volume and restrictive abnormalities. However, absolute Xrs and Rrs values should be evaluated carefully. It is well-known that Zrs values are affected by body size, specifically height, gender, and age, similar to those of the pulmonary function test (Oostveen et al. 2013). In our RA cohort, height significantly correlated with R4, R20, and R36 (Table 3). Although previous studies have tried to establish reference data from healthy subjects, standardized predictive values have not been established yet (Oostveen et al. 2003; Shiota et al. 2005; Oostveen et al. 2013). Moreover, the impedance data may vary between different FOT devices used for the measurements (Oostveen et al. 2013; Tanimura et al. 2014). Future studies are necessary to establish the methodology and reference values for Zrs measurements.

This study has several limitations. The data were retrospectively collected from RA patients with multiple respiratory disorders and different smoking statuses. Relatively high prevalence of obstructive abnormality (FEV_1_/FVC < 0.70) in the spirometry and emphysema in the CT findings suggests involvement of COPD. It is widely recognized that pulmonary manifestations in RA are heterogeneous (Tanaka et al. 2004). Although it is possible that a smoking history itself affects pulmonary functions and respiratory mechanics, pulmonary inflammation due to cigarette smoking is an important risk factor for developing RA specifically via anti-CCP antibody production (Klareskog et al. 2006). An association between positive anti-CCP antibody or RF and pulmonary complications of RA has been proposed (Klareskog et al. 2008; Reynisdottir et al. 2014). Consistent with those previous findings, the prevalence of anti-CCP antibody and RF was relatively high (88.2 and 86.3 %, respectively), most of which had abnormal findings on chest CT images. Therefore, effects of smoking cannot be ignored to understand clinical characteristics and pathophysiology of RA-related pulmonary diseases. Interestingly, there was no significant difference in the Zrs parameters (Rrs, Xrs and ΔXrs) between ever and never smokers due to large variability (Additional file 2: Figure S2). Bronde et al. reported that the prevalence of COPD and asthma in RA patients was 25 and 18 %, respectively, significantly higher than those without RA in a population-based study in Ontario, Canada (Brode et al. 2014). Taken together, our cases involving heterogeneous pulmonary abnormalities, different smoking statuses, and obstructive abnormality are likely to reflect a real-world clinical setting of patients with RA-related pulmonary diseases. Another limitation of the present study is that the healthy control subjects were not matched to the RA group and younger than RA patients. The Zrs results of our healthy subjects such as frequency-independent behavior in Rrs and within-breath behavior in Xrs (Fig. 2) are adequate as normal control data consistent with those in the previous reports (Kanda et al. 2010; Faria et al. 2012). However, prospective studies with a larger number of subjects including patients both with and without pulmonary abnormalities as well as healthy controls are necessary to characterize in more detail the respiratory impedance of RA.

## Conclusion

The respiratory mechanics together with pulmonary functions and CT findings were characterized in patients with RA-related airway and parenchymal abnormalities. Significant frequency-dependence in the Rrs parameters was found in the RA patients but not in the healthy subjects. It is likely that respiratory physiology of RA-related IP is different from those of idiopathic IP. Because impedance measurements are not invasive, FOT may become a useful tool to evaluate alterations in respiratory functions in RA patients in the future.

## Methods

### Subjects

Patients who met the 1987 American College of Rheumatology classification criteria for RA and attended the outpatient clinic of the Department of Respiratory Medicine, Nagoya University Hospital, between July 2010 and November 2012 were retrospectively reviewed. Sixty-nine patients on whom Zrs measurements, pulmonary function test, and CT examination had been performed were enrolled in this study. Healthy control data obtained from the hospital staff of our previous publication (Uchida et al. 2013) were re-analyzed for the impedance results (Additional file 3: Table S1).

### Pulmonary function tests

After impedance measurements, spirometry was performed and lung volumes were determined using computerized equipment (Fudak77, Fukuda Sangyo, Tokyo, Japan). The following spirometric parameters, vital capacity, FVC, FEV_1_, and FEF_25–75_, were measured. Lung volumes including residual volume and TLC were measured by means of the helium dilution technique. DL_CO_ and its value corrected for alveolar volume (DL_CO_/V_A_) were measured by the single-breath technique. Data were given as % of the predicted values for spirometry and lung volumes calculated according to the method of the Japanese Respiratory Society (Japanese-Respiratory-Society 2004).

### Respiratory impedance measurements

Impedance data was collected by FOT using a commercially available machine (MostGraph-01; Chest M.I., Tokyo, Japan) that generates a broad-band waveform at frequencies from 4 to 36 Hz in 4 Hz steps as described previously (Uchida et al. 2013). Briefly, impulse oscillatory signals generated by a loud speaker at intervals of 0.25 s were applied to the respiratory system during tidal breathing at rest. The Zrs was calculated using the system computer algorithms. The Zrs was recorded for approximately 20 s (5–6 respiratory cycles) while the patients firmly supported their cheeks with their palms in the sitting position using a nose clip with the neck in a comfortable neutral posture. Upper airway artifacts resulting from glottal changes, air leaks, and cheek support techniques during measurements significantly affect the impedance results (Peslin et al. 1985; Uchida et al. 2013; Bikov et al. 2015). Therefore, such upper airway artifacts were carefully eliminated. Three to five technically acceptable measurements were performed as recommended in the guidelines (Oostveen et al. 2003).

### Analysis of impedance results

The actual values of Rrs and Xrs at given frequencies between 4 and 36 Hz were analyzed in this study. Each impedance parameter was expressed as a mean value during a respiratory cycle, whole-breath, inspiration, and expiration. ΔXrs were calculated as mean inspiratory values minus mean expiratory values according to a method by Dellaca et al. (2004).

### Interpretation of CT examinations

CT data were obtained using a 64-row or 16-row multi-detector row CT (Aquilion64 or Aquilion16; Toshiba Medical Systems Corp., Tokyo, Japan). Patients were scanned in the craniocaudal direction with inspiratory apnea. The slice thickness and reconstruction interval of HRCT were 0.5-/1.0-mm and 0.5-/1.0-mm, respectively, using a high-spatial frequency algorithm. Routine CT (5-mm slice thickness and 5-mm interval) were also reconstructed. Both HRCT and routine CT were available for 66 cases (95.7 %), while only routine CT was available for 3 (4.3 %) cases. The radiological diagnosis was made by an experienced thoracic radiologist (S. Iw) and defined based on a previous report by Tanaka et al. (2004). The extent of CT findings was graded subjectively with a five-point scale within the whole lung field as follows: grade 0, the finding was absent; grade 1, the percentage of involvement of the lungs was between 1 and 25 %; grade 2, the percentage of involvement was between 26 and 50 %; grade 3, the percentage of involvement was between 51 and 75 %; and grade 4, the percentage of involvement was more than 76 % (Tanaka et al. 2004). He was blinded to the patients’ clinical information except that all patients had RA. Then, the diagnosis was reviewed by chest physicians (R.S and H.A.). Each CT finding was categorized as one of four types: airway lesion dominant, IP dominant, mixed (both airway lesion and IP) pattern, and others (Mori et al. 2012).

### Statistical analysis

Repeated-measure ANOVA followed by Bonferroni’s post hoc test or *t* test was used to evaluate the statistical significance (SigmaPlot11.0; Systat Software Inc., San Jose, CA). When data failed a normality test, ANOVA on ranks followed by a Tukey test or Mann–Whitney test was used. *P* < 0.05 was considered statistically significant. Correlations between valuables were analyzed using the Spearman’s rank or Pearson’s correlation coefficient. Fisher’s exact test was used to evaluate significance in group differences in various categories. Data were given as mean ± SD.

### Ethics, consent and permissions and consent to publish

This retrospective study was approved by the local ethics committee of Nagoya University Hospital (approval No. 2012-0352, 2015-0061). No patient identifiers were included. The informed consent requirement to participate and publish was waived for this retrospective analysis. The study information was disclosed to the target patients via the internet s at Nagoya University Hospital to allow the candidate patients to refuse to participate.

## Additional files

10.1186/s40064-016-1952-8 Representative CT images of airway lesion dominant and IP dominant patterns.
10.1186/s40064-016-1952-8 Respiratory impedance of smokers and never smokers.
10.1186/s40064-016-1952-8 Clinical characteristics of healthy control subjects.

## Abbreviations

ANOVA: analysis of variance
anti-CCP: anti-cyclic citrullinated peptide
COPD: chronic obstructive pulmonary disease
CT: computed tomography
Δ: the difference between inspiratory and expiratory phases
DL_CO_: diffusing capacity of the lung for carbon monoxide
FEF_25–75_: forced expiratory flow from 25 to 75 % of forced vital capacity
FEV_1_: forced expiratory volume in 1 s
FOT: forced oscillation technique
FRC: functional residual capacity
FVC: forced vital capacity
Rrs: respiratory resistance
R4: Rrs at 4 Hz
R20: Rrs at 20 Hz
R36: Rrs at 36 Hz
RA: rheumatoid arthritis
RF: rheumatoid factor
TLC: total lung capacity
V_A_: alveolar volume
VC: vital capacity
Xrs: respiratory reactance
X4: Xrs at 4 Hz
Zrs: respiratory impedance
