# Supplementary material for: Respiratory mechanics measured by forced oscillation technique in rheumatoid arthritis-related pulmonary abnormalities: frequency-dependence, heterogeneity and effects of smoking
Source: Springerplus. 2016 Mar 15;5:335. doi: 10.1186/s40064-016-1952-8 (PMC4792822; doi:10.1186/s40064-016-1952-8)

Additional file 1: Figure S1

Representative CT images of airway lesion dominant and IP dominant patterns.

A Airway lesion dominant

B Interstitial pneumonia dominant

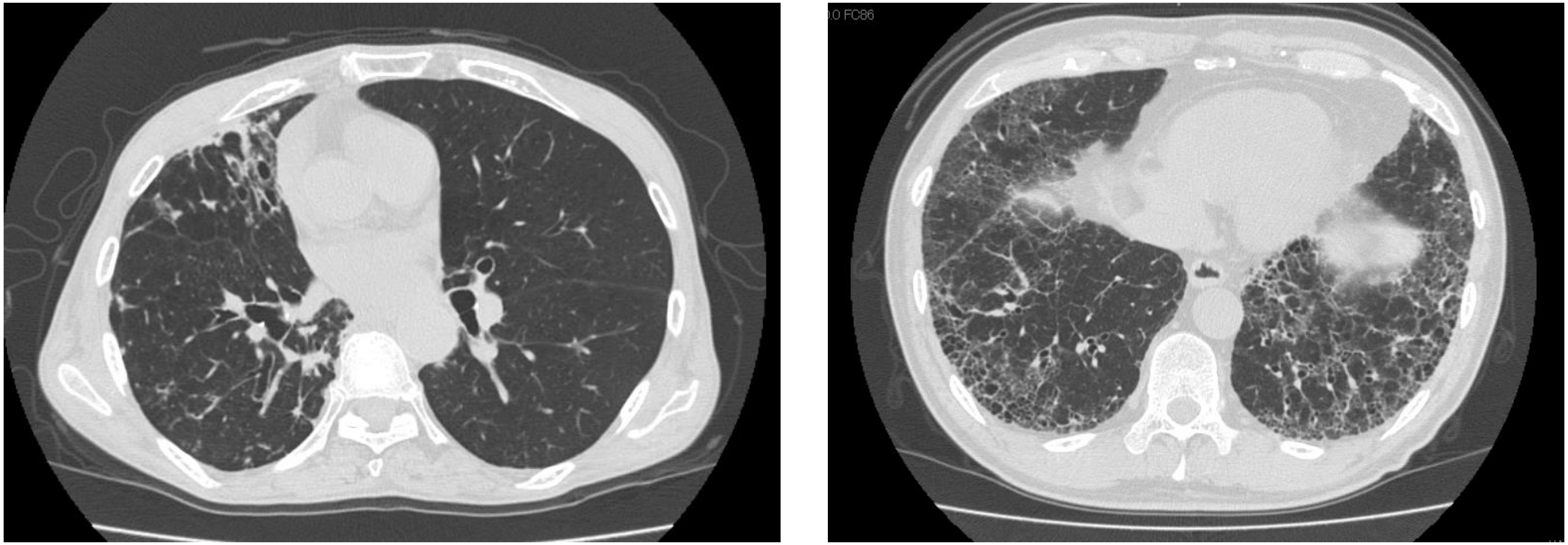

Supplement: Supplementary file 1 — 10.1186/s40064-016-1952-8 Representative CT images of airway lesion dominant and IP dominant patterns. [file 40064_2016_1952_MOESM1_ESM.pdf]
