# Supplementary figures and images for: Respiratory mechanics measured by forced oscillation technique in rheumatoid arthritis-related pulmonary abnormalities: frequency-dependence, heterogeneity and effects of smoking
Source: Springerplus. 2016 Mar 15;5:335. doi: 10.1186/s40064-016-1952-8 (PMC4792822; doi:10.1186/s40064-016-1952-8)

Additional file 2: Figure S2

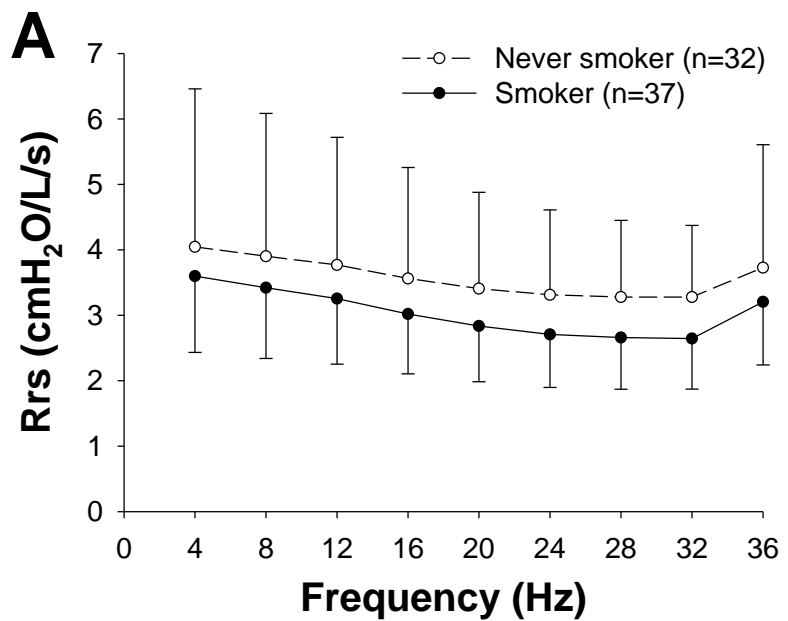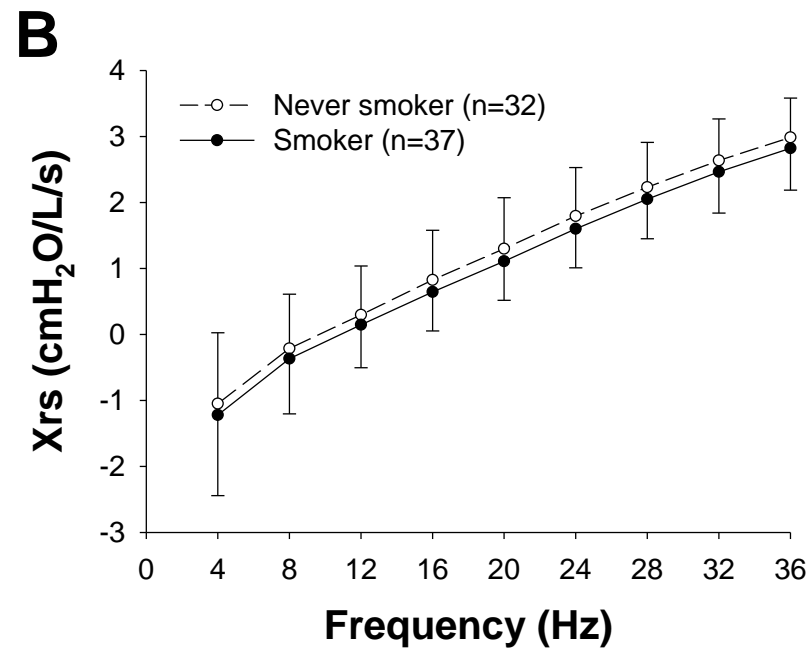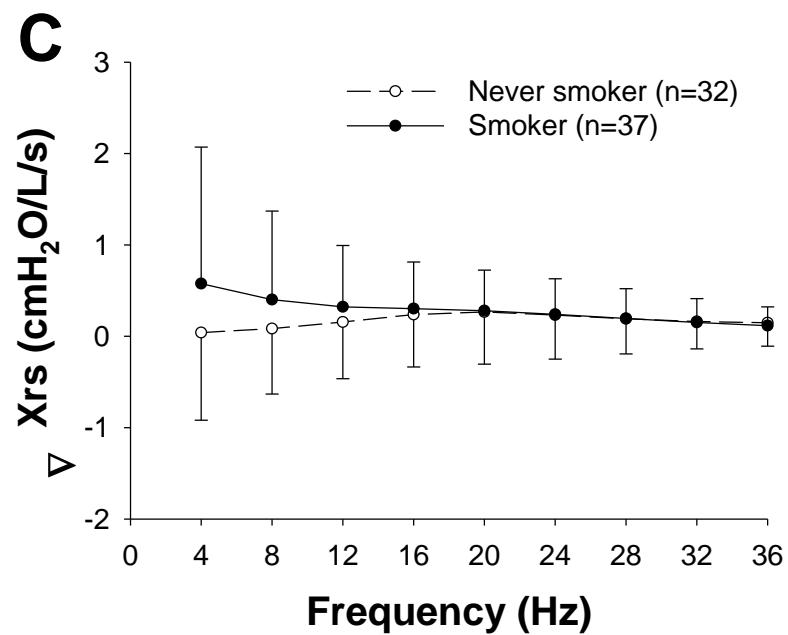

Supplement: Supplementary file 2 — 10.1186/s40064-016-1952-8 Respiratory impedance of smokers and never smokers. [file 40064_2016_1952_MOESM2_ESM.pdf]
