# Supplementary material for: Respiratory mechanics measured by forced oscillation technique in rheumatoid arthritis-related pulmonary abnormalities: frequency-dependence, heterogeneity and effects of smoking
Source: Springerplus. 2016 Mar 15;5:335. doi: 10.1186/s40064-016-1952-8 (PMC4792822; doi:10.1186/s40064-016-1952-8)
Supplement: Supplementary file 3 — 10.1186/s40064-016-1952-8 Clinical characteristics of healthy control subjects. [file 40064_2016_1952_MOESM3_ESM.pdf]

Additional file 3: Table S1.

Clinical characteristics of healthy control subjects

Subjects	Healthy (n=10)
Age, years (range)	38.4±9.6 (24-59)
Sex, male/female	4/6
Height, cm	164.0±9.3
Weight, kg	59.8±10.2
BMI	22.1±2.0
%VC	105.1±11.3
%FVC	108.2±11.0
%FEV_1	103.8±13.9
FEV_1/FVC, %	82.6±5.8
FEF_{25-75}/FVC, %	90.1±28.1
%FEF_{25-75}	89.6±30.7

Values are mean ± SD.
